# The Th17/Treg axis: a key to understanding and treating autoimmune disorders

**DOI:** 10.1515/biol-2025-1285

**Published:** 2026-03-05

**Authors:** Xiangrui Xie, Yang Liu, Huijing Li

**Affiliations:** College of Acupuncture and Massage, Changchun University of Chinese Medicine, Changchun, China; Acupuncture Department, Affiliated Hospital of Changchun University of Chinese Medicine, Changchun, China

**Keywords:** Th17 cell, treg cell, immune, autoimmune rheumatic diseases (ARDs), disease-modifying anti-rheumatic drugs (DMARDs)

## Abstract

In autoimmune rheumatic diseases (ARDs), T cells mistakenly attack the body’s own joints, skin, blood vessels, and other tissues, leading to chronic inflammation and tissue damage. Among these, the immune balance between T helper 17 lymphocytes (Th17) and regulatory T lymphocytes (Treg) is a foundation for maintaining normal immune function in the human body. An immune imbalance between Th17 and Treg cells is one of the key pathogenic mechanisms in ARDs. The percentages of Th17 and Treg cells can serve as important indicators for the severity of autoimmune diseases and treatment response. Therefore, by studying the origin and function of Th17 and Treg cells as well as the cytokine microenvironment that regulates their differentiation, we aim to modulate the immune state by restoring cellular balance. This approach is particularly relevant in ARDs such as rheumatoid arthritis, Sjögren’s syndrome, systemic lupus erythematosus, scleroderma, and ankylosing spondylitis. It also summarizes the current clinical application of disease-modifying anti-rheumatic drugs in regulating the balance between Th17 and Treg cells, with the aim of providing guidance for clinical practice.

## 1 Introduction

The innate and adaptive immune systems are the two divisions of the immune system [1]. T lymphocytes are a core component of adaptive immunity, responsible for cellular immune responses and immune regulation. One of the primary subgroups of T cells is CD4^+^ T lymphocytes. These lymphocytes can develop into regulatory T lymphocytes (Treg) and T helper 17 lymphocytes (Th17) according to their function. Th17 cells, also known as pro-inflammatory immune cells, have the ability to produce inflammatory cytokines and encourage the development of inflammation [2]; Treg cells are immune-suppressive cells that can secrete multiple anti-inflammatory factors to inhibit the progression of inflammation and induce the formation of immune tolerance [3]. Both cells are interconnected in their differentiation pathways and antagonistic in their functions, jointly participating in the regulation of immune responses [4]. For the body’s immune system to remain healthy, Th17/Treg cell balance is crucial. Inflammation, tumor growth, anti-parasitic immunity, organ transplant immunological rejection, and autoimmune disorders are all significantly influenced by Th17 cells [5]. Studies have demonstrated that the immunological balance between Th17 and Treg cells is crucial for the central nervous, respiratory, urinary, circulatory, and digestive systems [6]. Additionally, the pathophysiology of illnesses including osteoporosis, diabetic problems, the tumor microenvironment, and preterm delivery is also connected to the immunological balance between the two [7].

Excessive autoantibody synthesis, inflammatory cytokine release, and immune complex deposition cause autoimmune rheumatic diseases (ARDs) to develop [8]. ARDs encompass a variety of diseases that affect patients’ mobility and function, including rheumatoid arthritis (RA), Sjögren’s syndrome (SS), systemic lupus erythematosus (SLE), scleroderma (SD), and ankylosing spondylitis (AS). The development of ARDs, evaluation of disease activity, and development of therapy plans are all significantly impacted by the balance between Th17 and Treg cells. Together with inflammatory mediators such as interleukin (IL)-6 or IL-21, endogenous transforming growth factor beta (TGF-β) stimulates effector cell development during inflammatory reactions and autoimmune disorders. Subsequently, as levels of inflammatory mediators such as IL-6 decrease, TGF-β amplifies and maintains Treg cell function, thereby regulating effector cell activity to an appropriate level following treatment. The roles of Th17 and Treg cells in ARDs are currently poorly understood, and further research is needed to elucidate the complex processes behind the Th17/Treg cell imbalance and develop more effective treatment strategies that will improve the outcomes and quality of life of patients with ARDs.

The occurrence and progression of ARDs are attributable not only to genetic susceptibility but also to environmental factors. Among these, the commensal microbiota residing in barrier sites such as the gut and skin exert significant effects on the body’s immune regulation. Extensive research has indicated that specific microbial communities and their metabolites can directly modulate the functional differentiation of T cells and antigen-presenting cells, thereby regulating the body’s immune balance [9]. For example, patients with psoriasis exhibit elevated Firmicutes/Bacteroidetes ratios in their gut microbiota, reduced levels of beneficial bacteria such as *Akkermansia muciniphila*, and concurrent disruption of the intestinal barrier with bacterial translocation. These characteristics show significant positive correlations with skin Th17/Treg imbalance and systemic inflammatory responses [10]. RA models further confirm that intestinal filamentous bacteria promote autoantibody production and joint inflammation by inducing Tfh cell differentiation in Peyer’s patches and migrating to systemic lymph nodes, suggesting that the gut microbiota can regulate systemic autoimmunity [11]. Notably, microbiota–immune interactions extend beyond the gut: skin microbiomes similarly modulate local Th17 responses via pattern recognition receptors [12]. Collectively, these findings elucidate the critical role of the microbiome as a core environmental regulator in maintaining Th17/Treg homeostasis. They provide a robust theoretical foundation for reshaping immune tolerance and treating autoimmune diseases through targeted microbiome therapies such as dietary intervention, probiotic supplementation, or fecal microbiota transplantation.

## 2 Th17/Treg cell balance and related pathways

### 2.1 Th17/Treg cell balance

**Figure 1:**
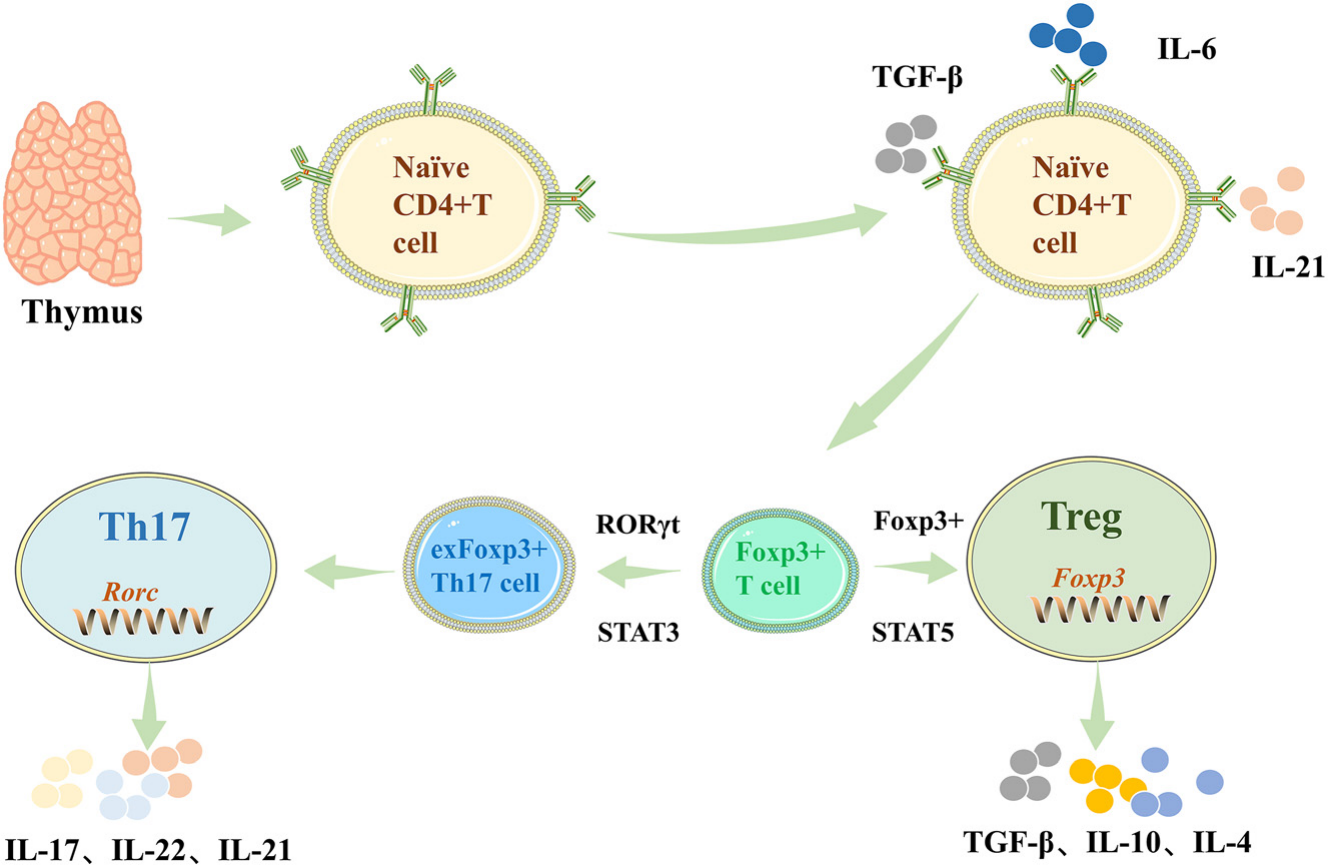
The differentiation process of Th17/Treg. Lymphoid progenitor cells differentiate in the thymic microenvironment, migrate out of the thymus into the peripheral circulation, and become naive T cells. Under the influence of TGF-β, IL-6 and IL-21 cytokines, they differentiate into Foxp3^+^ T cells. Mediated by RORγt and STAT3 molecules, Foxp3^+^ T cells can be converted into exFoxp3^+^ Th17 cells. Th17 cells and exFoxp3^+^ Th17 cells aggregate and produce IL-17, IL-22, and IL-21. Under the mediation of Foxp3 and STAT5 molecules, Foxp3^+^ T cells differentiate into Treg cells, thereby secreting TGF-β, IL-10, and IL-4.

Multipotent hematopoietic stem cells (HSCs) in the bone marrow are the source of T cells. After HSCs differentiate into lymphoid progenitor cells, they migrate to the thymus, where they survive selection in the thymic microenvironment and differentiate into CD4^+^ or CD8^+^ single-positive cells. Based on T cell receptor chains, surface markers, secreted factors, and functional characteristics, mature T cells can be classified into several major subsets, including CD4^+^ helper T cells, CD4^+^ Treg cells, CD8^+^ cytotoxic T cells, γδ T cells, natural killer T cells, and mucosa-associated constant T cells possessing innate immune properties. Th17 cells are a pro-inflammatory T cell subset characterized by the secretion of cytokines from the IL-17 family, but they can also secrete other cytokines such as IL-21 and IL-22. They play a central role in mediating host defense and inflammatory diseases [13]. Conversely, Treg cells are primarily responsible for maintaining immune tolerance by secreting anti-inflammatory factors such as IL-10 and TGF-β to suppress excessive immune responses [4], 8]. The differentiation balance between Th17 and Treg cells is precisely regulated by the cytokine environment, with TGF-β serving as a common foundational factor essential for both differentiation pathways. The specific direction is determined by other inflammatory signals: in the presence of TGF-β alone, naive CD4^+^ T cells tend to differentiate into Treg cells; when inflammatory factors such as TGF-β, IL-6, or IL-21 coexist, the differentiation pathway shifts toward Th17 cells, suppressing Treg generation (Figure 1). At the transcriptional level, Th17 cells specifically overexpress the nuclear transcription factor retinoic acid-related orphan receptor gamma t (RORγt) [14], which drives the production of effector molecules such as IL-17 [15], recruits neutrophils, and promotes inflammation [16]. In contrast, Treg cells specifically express the key transcription factor forkhead box protein p3 (Foxp3) [17]. This protein not only serves as a hallmark feature of Treg cells but also maintains immune homeostasis by stabilizing their suppressive phenotype and inhibiting their transdifferentiation into pro-inflammatory subsets such as Th17 cells [18]. Research has further elucidated the molecular mechanisms underlying cytokine regulation: TGF-β signaling enhances Foxp3 acetylation, thereby promoting its transcriptional suppression function; conversely, inflammatory signals such as IL-6 reverse this process, weakening Treg cells’ inhibitory capacity. The synergistic action of TGF-β and IL-6 also promotes Th17-associated transcriptional programs through mechanisms including the upregulation of ubiquitin-specific protease 4 [19]. Thus, Th17 cells drive inflammation by secreting pro-inflammatory factors, while Treg cells exert suppression by releasing anti-inflammatory factors. The dynamic equilibrium between these two functions is crucial for immune homeostasis. Disruption of this balance is closely associated with the onset and progression of various autoimmune diseases and chronic inflammatory conditions, including RA and SLE [5].

### 2.2 Signaling pathways related to immune balance

**Figure 2:**
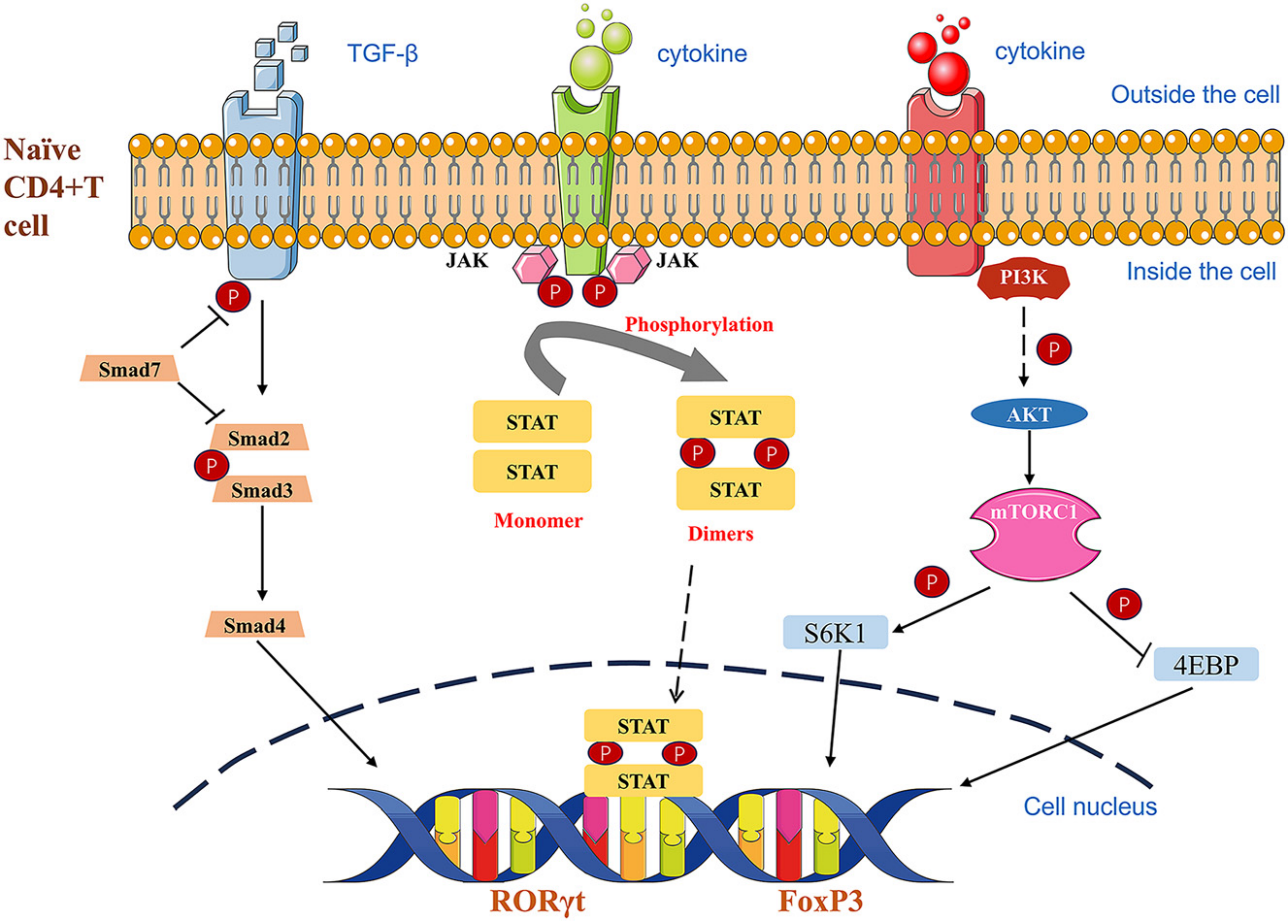
Signaling pathways involved in Th17/Treg cell differentiation and cytokine release.

An increasing number of scholars have begun to study the signaling pathways or cytokines that cause Th17/Treg cell imbalance, including the Janus kinase (JAK)/signal transducers and activators of transcription (STAT) signaling pathway, phosphatidylinositol 3-kinase (PI3K)/protein kinase B (also known as AKT)/mammalian target of rapamycin (mTOR) signaling pathway, and TGF-β/Smad signaling pathway (Figure 2). Researchers can better comprehend human rheumatic immune diseases and identify therapeutic targets for medication treatment of immunological disorders by doing in-depth studies on Th17/Treg cell imbalance.

#### 2.2.1 JAK/STAT signaling pathway

The pathophysiology of autoimmune disorders and inflammation is linked to the JAK/STAT signaling pathway. Cell surface receptors, JAK proteins, and STAT proteins are the three parts of this signaling pathway. The non-receptor tyrosine kinases JAK1, JAK2, JAK3, and TYK2 are members of the JAK family. The main activation factors are STAT1 and STAT3 [20]. Cytokines attach to transmembrane receptors in this pathway, triggering receptor-associated JAKs that phosphorylate intracellular JAK proteins [21], 22]. This consequently leads to the activation, phosphorylation, and dimerization of STAT molecules. These dimers control gene transcription after being delivered to the cell nucleus [23], [24], [25]. One important molecular pathway that starts CD4^+^ T cells’ differentiation into particular T cells is the JAK/STAT pathway. IL-6 and IL-23 can increase the proportion of Th17 cells by inducing RORγt expression through the STAT3 pathway while inhibiting Foxp3 expression, thereby suppressing Treg cell differentiation [26]. STAT5 enhances the differentiation of Treg by modulating FoxP3 expression [27]. The initiating factors for the JAK/STAT signaling pathway include not only cytokines but also growth factors such as ILs and interferons [20]. JAK3 can be activated by some cytokines that have a common *γ* chain, including IL-2, IL-4, IL-7, and IL-21. Cytokines with gp130 subunit ligands can bind to JAK1, JAK2, and TYK2 isoforms [28]. Th0 cells can differentiate into Th17 cells with the help of TGF-β and IL-6 via the JAK/STAT3 signaling pathway [29]. In addition to stimulating the JAK/STAT3 signaling pathway and upregulating the expression of the transcription factors RORγt and RORα, IL-23 promotes Th0 cells to develop into Th17 cells [30]. Additionally, cytokines such as IL-2, IL-7, IL-4, and IL-12 negatively regulate Th17 cell differentiation by activating the JAK/STAT pathway [31], thereby modulating the Th17/Treg balance. Furthermore, STAT3 regulates Th17 cell differentiation by directly acting on the genes encoding IL-17a–f, ROR, and IL-23 receptors as well as other genes that promote Th17 cell differentiation [32]. Experiments have shown that J069, as a JAK/STAT inhibitor, effectively reduces STAT3 expression; inhibits Th1, Th2, and Th17 differentiation; and promotes Treg differentiation, demonstrating potential for treating immune-related diseases. Since T cell surface cytokine receptors lack enzymatic activity, they must bind to JAK proteins to interact with corresponding cytokines and initiate T cell differentiation [33]. Therefore, one important mechanism that mediates CD4^+^ T cell differentiation and maturation is the JAK/STAT signaling system.

#### 2.2.2 PI3K/Akt/mTOR signaling pathway

The PI3K/AKT/mTOR signaling pathway plays a crucial role in altering the differentiation state of CD4^+^ T cells, inhibiting Th17 cells, and promoting the differentiation of Treg cells. This signaling pathway is a highly organized and complex intracellular signaling network, with its core components including PI3K, AKT, and mTOR. A key factor in T cell differentiation is mTOR [34]. A protein kinase in the PI3K signaling pathway that lymphocytes activate, mTOR is involved in cell proliferation and differentiation and is essential in regulating the proportion of Treg cells to Th17 cells [35]. mTOR controls the fate of various effector T cells, including Th1, Th2, and Th17 cells [36]. According to studies, Th17 cell differentiation is impacted both *in vitro* and *in vivo* by p85a knockout, PI3K/mTORC1 inhibitor use, and raptor knockout to inhibit AKT activity. Consequently, less IL-17A and IL-17F are produced [37]. Previous studies have confirmed that AKT Ser473 phosphorylation and AKT Thr308 phosphorylation have different substrates, so AKT has two main pathways: one is involved in Th17 cell regulation, while the other participates in Treg cell differentiation [38]. Mechanistic target of rapamycin complex (mTORC) 1 has been shown to regulate the activation of STAT3 and STAT5, with the mechanism being that mTORC1 can inhibit the expression of suppressor of cytokine signaling 3, suggesting that the equilibrium between Th17 and Treg cells may be influenced by mTORC1 [39]. One of the most extensive cell-related routes is the PI3K/AKT/mTOR signaling system, which controls cellular functions such as metabolism, survival, differentiation, and proliferation. Tumors, autoimmune disorders, and fibrotic diseases are among the conditions that exhibit abnormal expression of this pathway [40].

#### 2.2.3 TGF-β/Smad signaling pathway

The TGF-β/Smad signaling pathway, also known as the receptor-coupled serine/threonine kinase signaling transduction pathway, currently has three subtypes of TGF-β identified in mammals: TGF-β1, 2, and 3, with TGF-β1 being the most highly expressed [41]. The type I TGF-β receptor is activated when activated TGF-β1 interacts with the type II TGF-β receptor. Both type I and type II serine/threonine kinase receptors mediate Smad signal transduction [42]. The Smad family serves as intracellular messengers in TGF-β signal transduction [43]. Studies have shown that Treg cells can secrete large amounts of TGF-β, exhibiting anti-inflammatory functions. TGF-β signaling can exert biological effects through its downstream Smad-dependent and Smad-independent pathways [44]. Additionally, TGF-β plays a vital role in mediating interactions between Th17 and Treg cells by triggering the STAT3 pathway, which can trigger the production of the transcription factors RORγt and Foxp3 [45].

## 3 Th17/Treg balance and ARDs

Research on the Th17/Treg immunological balance has steadily gained popularity due to its strong correlation with the initiation and progression of ARDs. Because these cells can release numerous cytokines that trigger additional inflammatory reactions and cause autoimmune disorders to gradually develop, they are important in ARDs. The findings are presented in two aspects: clinical studies (Table 1) and experimental studies (Table 2).

**Table 1:** Clinical research on Th17/Treg cell balance in autoimmune rheumatic diseases.

Disease	Target points	Mechanism	Ref.
RA	TGF-β1; IL-10	Secreting inflammatory factors, synovial Tregs suppress inflammation.	[52]
RA	STAT3; STAT5	Knocking out STAT3 in synovial cells reduces Th17 content and increases Treg content, while knocking out STAT5 has the opposite effect.	[53], 54]
RA	IL-6; IL-17; TGF-β; IL-10	By modulating inflammatory markers, actively regulating RORγt and Foxp3, thereby maintaining homeostasis.	[55]
RA	IFN-γ; IL-4; IL-17A; IL-22; IL-10	Elevated levels of IFN-γ, IL-4, IL-17A, and IL-22, coupled with decreased IL-10 levels, thereby maintaining homeostasis.	[56]
RA	SMAD3; SMAD4; STAT3; STAT5	MicroRNAs regulate gene expression of transcriptional factors, which influence Th17/Treg balance.	[57]
RA	RORγt; Foxp3	Regulate the mRNA expression of transcription factors RORγt and FoxP3.	[58]
pSS	IFN-γ; IL4; IL-17; IL-10; RORγt; Foxp3	Th17/Treg polarization imbalance, with Th17 cells predominating.	[66]
pSS	IFN-γ; TNF-α; IL-6; IL-17A; IL-17F; IL-10; TGF-β	Inhibiting IFN-γ, TNF-α, IL-6, IL-17A, and IL-17F secretion, and promoted IL-10 and TGF-β secretion.	[67]
pSS	Foxp3	Polymorphisms in Foxp3 and CTLA-4 genes were associated with the susceptibility to pSS.	[68]
pSS	IL-2	IL-2 inhibits Th17 differentiation in a Treg-independent manner.	[69]
SLE	IL-6;IL-17	IL-6 promotes the generation of Th17 cells.	[75]
SLE	miR-16-5p/LATS1	miR-16-5p targets and inhibits LATS1, thereby restoring the Th17/Treg balance.	[76]
SLE	miR-1205/FoxP3	Inhibiting miR-1205 restores Treg function and reverses the progression of SLE.	[77], 78]
SLE	miR-19b	miR-19b targets and suppresses KLF13, restoring the Th17/Treg balance.	[79]
SLE	IL-23; IL-2	Decreased IL-23, increased IL-2 production, decreased IL-17 production.	[81]
SSc	RORC	The conversion of Tregs to Th17 cells leads to immune imbalance.	[91]
SSc	IL-17A; Foxp3	High expression of IL-17A and Foxp3 inactivation lead to an imbalance between Th17 and Treg cells.	[92]
AS	IL-17; TNF-α; IL-6; IL-10	Elevated levels of IL-17, TNF-α, IL-6, and Th17, with no changes in Treg and IL-10 levels.	[103]
AS	IFN-γ; IL-17A	By increasing IFN-γ and IL-17A expression, it exacerbates inflammation and disrupts the Th17/Treg balance.	[105]
AS	TNF-α; IL-6; IL-17; IL-23; TGF-β	Restore balance by downregulating Th17/proinflammatory factors and upregulating the Treg/TGF-β axis.	[106]
AS	RORγt; Foxp3; IL-17; IL-22	By enhancing RORγt expression and reducing Foxp3 expression, it simultaneously increases the expression and secretion of IL-17 and IL-22.	[107]
AS	IL-10	Tregs suppress Th17 cells by secreting IL-10, thereby promoting new bone formation.	[108]

**Table 2:** Experimental studies on Th17/Treg cell balance in autoimmune rheumatic diseases.

Disease	Study subjects	Intervention	Target points	Mechanism	Ref.
RA	collagen-induced arthritis (CIA) mouse	Wutou decoction	JAK2/STAT3 pathway	Inhibiting JAK2/STAT3 phosphorylation, thereby modulating the stability of Treg cells and the Treg/Th17 balance.	[59]
RA	collagen-induced arthritis (CIA) mouse	Duanteng-Yimu Tang (DTYMT)	RORγt; Foxp3	Inhibiting Th17 cell differentiation and promoted Treg cell production, thus improving the Treg/Th17 imbalance.	[60]
pSS	NOD mice	LGMSC-Exos	IL-17; IFN-γ; IL-6; TGF-β; IL-10	It downregulates IFN-γ, TNF-α, IL-6, and IL-17, upregulates TGF-β and IL-10, and suppresses autoimmune reactions by promoting Tregs and inhibiting Th17 cells.	[70]
pSS	NOD/Ltj mouse	B7-H4Ig	IL-12; IL-6; IL-18; IL-1α; TNF-α; IFN-α	It reduces the levels of IL-12, IL-6, IL-18, IL-1α, TNF-α, and IFN-α, thereby inhibiting Th17 cell differentiation.	[71]
SLE	Mice	/	RORγt; Foxp3	Reduce IL-17 secretion, decrease RORγt expression, and increase Foxp3 expression.	[80]
SLE	Mice	/	IL-23; IL-2	Decreased IL-23, increased IL-2 production, decreased IL-17 production.	[81]
SLE	Mice	/	STAT3	STAT3 deficiency specific to Tregs enhances Th17-mediated inflammation.	[82]
SLE	C57BL/6 mice	HSD	TGF-β; IL-17	Increase TGF-β and IL-17, upregulate Th17, and exacerbate inflammation.	[83]
SSc	Mice	/	Gut microbiota	Enhancing Tregs to Modulate Th17/Treg Imbalance.	[93]
SSc	BALB/c mice	Thalidomide	IL-17A; Foxp3; TGF-β1/Smad3	Inhibits the TGF-β1/Smad3 pathway, downregulates Th17/IL-17A and upregulates Treg/Foxp3, thereby correcting the Th17/Treg imbalance.	[94]
SSc	C57BL/6 mice and IL-21 knockout mice	/	IL-21; STAT3	IL-21 contributes to the development of SSc by promoting the expression of fibrosis-related genes and modulating the levels of CD4^+^ T cells.	[95]
SSc	knockout mice	/	TLR7; TLR9	TLR7 deficiency shifts the Th17/Treg balance toward anti-inflammatory and anti-fibrotic effects by regulating pDCs, whereas TLR9 deficiency has the opposite effect.	[96]

### 3.1 RA

The symptoms of RA, a complicated autoimmune disease, include joint discomfort, swelling, and increasing deterioration. The fundamental pathological changes include synovitis and vascular proliferation [46], 47], with T lymphocyte infiltration being a key mechanism in its pathogenesis. The global prevalence of RA is 1–2 % [48]. Therefore, the disruption of Th17/Treg balance is considered one of the key mechanisms underlying the onset and chronic inflammation of RA [49], [50], [51].

Research has indicated that the synovial tissue of patients with RA contains Treg cells with compromised regulatory function. Treg cells can secrete anti-inflammatory chemicals such as TGF-β1 and IL-10, which have a negative regulatory influence on inflammation [52]. In the synovial cells of patients with RA, knocking out STAT3 has been shown to decrease Th17 levels and raise Treg levels, while knocking out STAT5 yields the opposite effect [53]. Furthermore, studies have demonstrated that SMAD3 and STAT3 exhibit a positive correlation in these patients [54]. Additionally, yoga helps maintain Th17/Treg cell homeostasis in patients with RA by actively modulating RORγt and Foxp3 through the regulation of inflammatory markers such as IL-6, IL-17, TGF-β, and IL-10, thereby sustaining homeostasis [55]. Patients with RA exhibit elevated levels of interferon-gamma (IFN-γ), IL-4, IL-17A, and IL-22, alongside decreased levels of IL-10 [56]. The microRNA expression profile influencing Treg/Th17 cell balance in patients with RA correlates with the expression of selected transcription factors such as SMAD3, SMAD4, STAT3, and STAT5 [57]. These patients also exhibit increased peripheral Th17 cell and RORγt expression but reduced Treg cell and Foxp3 expression [58]. Research has indicated that Chinese herbal decoctions alleviate arthritis inflammation in collagen-induced arthritis (CIA) mice by regulating Treg cell stability and Treg/Th17 balance by inhibiting JAK2/STAT3 phosphorylation [59]. In particular, Duanteng-Yimu Tang alleviates joint damage in CIA mice. Its regulation of RORγt and Foxp3 mirrors the previous experiment while simultaneously reducing IL-1β, IL-17, and tumor necrosis factor (TNF)-α mRNA levels and increasing IL-10 mRNA levels, thereby correcting the Treg/Th17 imbalance [60].

### 3.2 SS

Immune cell infiltration is a hallmark feature of SS, a chronic autoimmune disease that gradually damages the lacrimal and salivary glands [61]. Principal Sjögren’s syndrome (pSS) and secondary Sjögren’s syndrome (sSS) are the two principal classifications of SS. pSS is a chronic autoimmune disease primarily affecting the exocrine glands, causing chronic inflammatory reactions in the submandibular glands, with extensive infiltration of lymphocytes, inflammatory factors, and immune complexes in the glands. It may even involve other systems and does not coexist with other rheumatic immune diseases. sSS refers to SS that develops on the basis of rheumatic immune diseases such as RA or SLE [62]. Its pathogenesis is more complex, and treatment is more challenging. Currently, the prevalence of pSS varies from 0.01 % to 0.77 % worldwide [63], with a higher prevalence in women and a peak incidence at the age of 50 years [64]. The pathophysiology of autoimmune diseases is significantly influenced by Th17 and Treg cells [65].

*In vitro* experiments on monocyte-derived dendritic cells from patients with pSS have revealed an imbalance in Th17/Treg polarization among CD4 T cell cytokines (IFN-γ, IL-4, IL-17, and IL-10) and transcription factors (RoRγt and Foxp3), with Th17 cells predominating [66]. The immunomodulatory effects of umbilical mesenchymal stem cell-derived exosomes reduce the Th17/Treg ratio in patients with pSS; suppress IFN-γ, TNF-α, IL-6, IL-17A, and IL-17F secretion; promote IL-10 and TGF-β secretion; and restore Th17/Treg balance via autophagy pathways [67]. In the peripheral blood leukocytes of patients with pSS, polymorphisms in the Foxp3 and cytotoxic T lymphocyte-associated antigen-4 (CTLA-4) genes are associated with susceptibility to pSS [68]. Compared with healthy individuals, patients with SS exhibit significantly increased Th17 cell levels in the salivary glands and serum, with no significant changes in Treg cell levels [69]. In experiments using non-obese diabetic (NOD) mice and peripheral blood mononuclear cells from patients with SS, it has been shown that labial gland-derived mesenchymal stem cells and their exosomes inhibit Th17 cell differentiation and promote Treg cell proliferation by decreasing IL-17, IFN-γ, and IL-6 levels while increasing TGF-β and IL-10 secretion [70]. Specific monoclonal antibodies blocking endogenous B7-H4 can promote T cell responses. In NOD/Ltj mouse pSS models, B7-H4Ig promotes Treg expansion, enhances the Th17/Treg balance, and reduces lymphocyte infiltration into the salivary glands [71]. Regulating the Th17/Treg immune balance and restoring immune homeostasis are crucial for treating SS.

### 3.3 SLE

SLE is a chronic inflammatory autoimmune disease characterized by fever, fatigue, chronic pain, photosensitivity, and skin rashes [72]. The annual incidence of SLE worldwide is 5.14 per 100,000, and the prevalence is 43.7 per 100,000 [73]. The pathogenesis of SLE involves genetic and environmental factors as well as innate and adaptive immune dysfunctions, leading to increased apoptosis frequency and reduced efficiency in clearing apoptotic debris, abnormal differentiation and activation of immune cells, and the production of excessive antibodies, ultimately causing damage to multiple organs [74].

Patients with SLE have higher levels of IL-6 and IL-17, which may indicate that IL-6 stimulates the production of Th17 cells [75]. The Th17/Treg imbalance in SLE correlates with reduced miR-16-5p expression in CD4^+^ T cells. Adipose-derived stem cell exosomes deliver miR-16-5p, which targets and inhibits large tumor suppressor homolog 1, thereby restoring Th17/Treg balance [76]. In SLE, circETS1 inhibits Treg differentiation via the miR-1205/FoxP3 axis, leading to Th17/Treg imbalance [77], 78]. Umbilical cord blood mesenchymal stem cell exosomes delivering miR-19b target Kruppel-like factor 13 inhibition, restoring Th17/Treg balance and reducing inflammation in patients with SLE [79]. The basophils of patients with SLE promote B cell production of autoantibodies and Th17 cell differentiation. MRL-lpr/pr mice lacking basophils exhibit significantly reduced serum IL-17 levels, indicating that activated basophils promote IL-17 production [80]. Reduced IL-2 levels and aberrant anti-dsDNA antibody production may result from increased levels of IL-23 and its receptor among patients with SLE. MRL-lpr mice with IL-23 receptor defects demonstrate reduced lupus nephritis symptoms, increased IL-2 production, and reduced IL-17 production [81]. High-salt diets in lupus mice increase disease severity and reduce survival rates, which are linked to notable elevations in Th1 and Th17 cell levels as well as in the Th17/Treg ratio in lupus mice [82]. Additionally, *in vitro* studies have found that high salt levels upregulate Th17 cells, while serum and glucocorticoid-regulated kinase 1 (SGK1) inhibitors can reverse the effects of high-salt treatment on Th17 cells. In addition, SGK1 phosphorylation targets forkhead box protein O1 and decreases its activity, which promotes RORγt-mediated transcription of IL-23R, increases IL-23R expression, and stimulates Th17 cell development both *in vitro* and *in vivo* [83].

### 3.4 SD

SD is primarily characterized by fibrosis and hardening of the skin and organ tissues [84]. It is mainly divided into two types: localized scleroderma (LS) and systemic sclerosis (SSc). LS primarily affects the skin, manifesting as localized skin thickening and hardening, which may also affect the joints and muscles [85]. More extensive skin and internal organs, such as the digestive system, kidneys, and lungs, are affected by SSc, which can result in organ malfunction in extreme circumstances. The average disease duration for SSc is 11.7 years [86], and it has the highest case-specific mortality rate among autoimmune diseases, with 50 % of patients dying directly from the disease [87]. In the early stages of SD, there is a decrease in the quantity of functional Treg cells and a notable infiltration of CD4^+^ T lymphocytes together with associated cytokines and chemokines in the blood and skin [88]. According to studies, 60 % of early diffuse-type SSc skin lesions include RNA sequencing of CD4^+^ T cells, suggesting that immunological responses are important in the early stages of the disease [89]. Additionally, research has found that IL-4 can induce multiple cells, including T lymphocytes, to produce TGF-β [90], further exacerbating tissue fibrosis.

In patients with SSc, Treg and Th17 cells undergo synchronous expansion, but Treg cells exhibit impaired suppressive function and high RORC gene methylation. The conversion of Treg cells to Th17 cells leads to immune imbalance [91]. Patients with SSc demonstrate increased Th17 cells with high IL-17A expression; Treg levels remain normal, but function is impaired, with Th17/Treg imbalance contributing to fibrosis progression [92]. *Heligmosomoides polygyrus* infection improves SSc skin fibrosis by inducing Treg cells, suppressing Th17 cells, and modulating the gut microbiota [93]. Thal inhibits the TGF-β1/Smad3 pathway and downregulates Th17/IL-17A while upregulating Treg/Foxp3, correcting the Th17/Treg imbalance in SSc, and alleviating skin and lung fibrosis [94]. IL-21 promotes the expression of fibrosis-related genes in skin fibroblasts by activating STAT3 while increasing Th1/Th2/Th17 frequencies and the Th17/Treg ratio, thereby driving skin and lung fibrosis in SSc [95]. In SSc, Toll-like receptor (TLR) 7 deficiency shifts Th17/Treg balance toward anti-inflammatory and anti-fibrotic effects via plasmacytoid dendritic cells, reducing skin and lung fibrosis; conversely, TLR9 deficiency exacerbates fibrosis. TLR7 promotes inflammation and fibrosis, while TLR9 exerts protective regulatory effects [96].

### 3.5 AS

AS presents early with mild low back pain or morning stiffness. As symptoms worsen, they may spread from the lumbar spine to the thoracic and cervical spines, leading to spinal deformity and ankylosis in severe cases, with a risk of disability. Epidemiological studies have indicated that the prevalence of this disease in young adults is approximately 0.3 % [97], with a higher incidence among male individuals aged 13–31 years [98]. Current research has suggested that AS may be an inflammatory disease caused by immune imbalance [99]. The imbalance between Th17 and Treg levels is a key element in the pathophysiology of AS [100]. Th17 cells’ IL-17 cytokine has the ability to trigger the production of inflammatory cytokines including IL-6, IL-1, and TNF by the innate immune system. These inflammatory factors, along with IL-17, accumulate in synovial fibroblasts, increasing osteoclast expression [101]. In a clinical study, the proportion of Treg cells in the peripheral blood of patients with AS was significantly smaller, while the proportion of Th17 cells was elevated [102].

These findings demonstrated that Th17 levels as well as IL-17, TNF-α, and IL-6 levels were significantly elevated in patients with AS compared with healthy individuals, while Treg and IL-10 levels showed no significant differences [103]. Additionally, IL-17A has been shown to be primarily responsible for the pathophysiology of AS [104]. Patients with AS exhibit elevated proportions of dual TCR T cells in their synovial fluid, with pTh17 and Treg cells showing clonal expansion and overexpression of characteristic transcription factors. Overlapping TCR repertoires exist between these subsets, enabling mutual conversion and participation in inflammatory regulation [91]. The increased Th17/Treg ratio in patients with AS exacerbates inflammation by amplifying IFN-γ and IL-17A expression, correlating positively with disease severity [105]. Anti-TNF-α therapy in AS reduces Th17 levels and increases Treg levels in responders, with opposite effects in non-responders. The mechanism involves downregulating Th17/pro-inflammatory factors and upregulating the Treg/TGF-β axis [106]. Elevated serum semaphorin 4D levels in patients with AS activate the aryl hydrocarbon receptor pathway via the CD72 receptor, promoting Th17 differentiation while suppressing Treg cells and exacerbating inflammation [107]. In AS, Treg cells inhibit Th17-mediated bone formation by secreting IL-10; the decreased Th17/Treg ratio reflects this process [108].

## 4 Drugs related to the Th17/Treg axis

**Figure 3:**
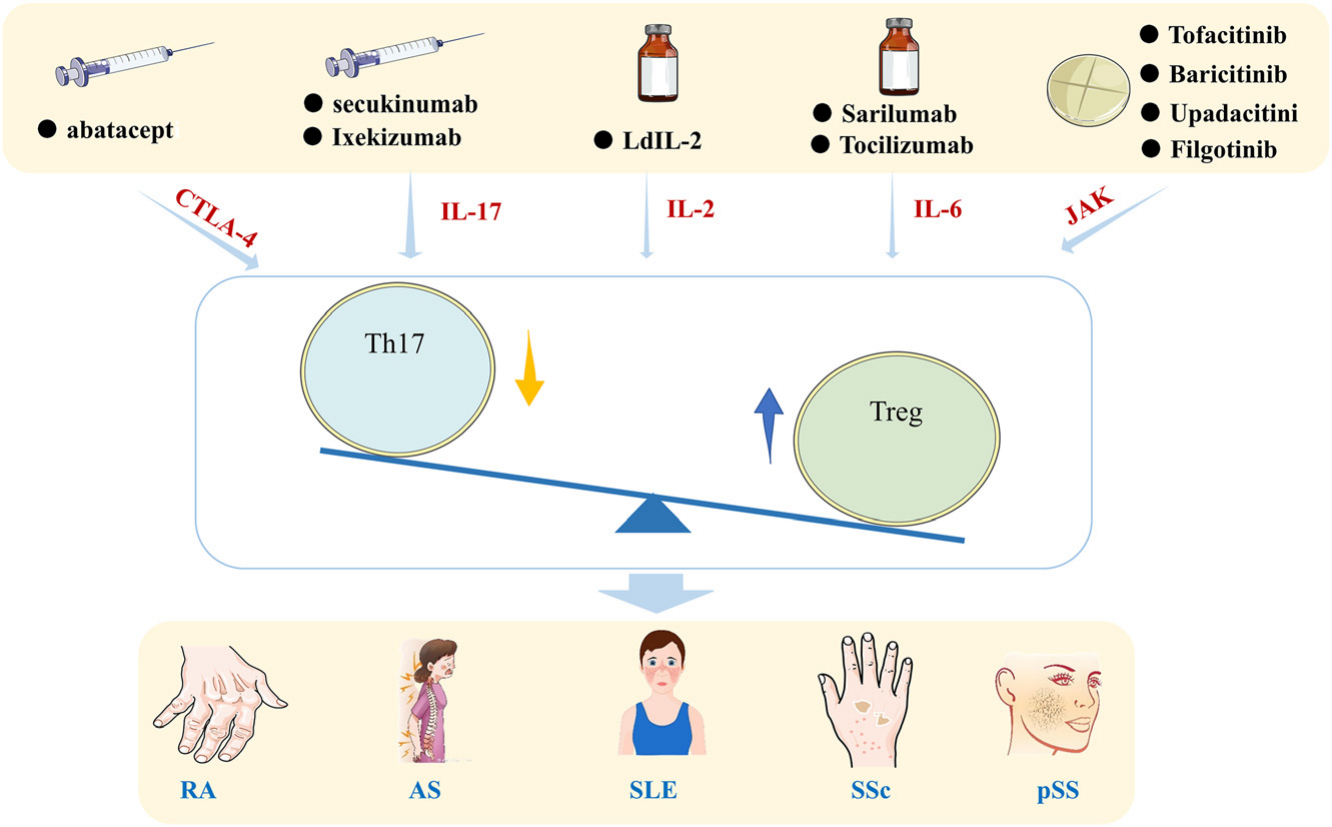
Application of Th17/Treg cell balance in DMARDs.

Disease-modifying anti-rheumatic drugs (DMARDs) are a class of medications used to treat rheumatic and immune-mediated diseases. Numerous drugs modulate Th17 and Treg cells, demonstrating clinical efficacy in ARDs. This analysis summarizes therapeutic agents targeting Th17/Treg balance (Figure 3 and Table 3), providing novel strategies for clinical drug development and immunology-related research.

**Table 3:** Clinical application of Th17/Treg cell balance in DMARDs.

Therapeutic strategy	Mechanism	Treatment methods	Disease	Ref.
Low-doses IL-2	Restoring immune tolerance through selective amplification of regulatory T cells.	Low-dose IL-2	RA	[111], [112], [113, 120]
			pSS	[114], [115], [116]
			SLE	[117], [118], [119], [120]
			AS	[120]
IL-17/IL-17R Inhibitors	By neutralizing IL-17A to block the inflammatory pathway	Secukinumab	RA	[131], 132]
			AS	[134], 135]
			SLE	[131]
			SSc	[133]
		Ixekizumab	RA	[131]
			SLE	[131]
IL-6/IL-6R Inhibitors	IL-6/IL-6R inhibitors broadly suppress inflammatory responses, immune cell activation, and autoantibody production by specifically blocking IL-6-mediated signaling pathways.	Tocilizumab	RA	[146], 154]
			AS	[150]
			pSS	[144], 147], 148]
			SLE	[134], 149]
			SSc	[151], 152]
		Sarilumab	RA	[154]
JAK inhibitors	By blocking the intracellular JAK-STAT signaling pathway, it inhibits inflammation and immune responses mediated by various pro-inflammatory cytokines.	Tofacitinib	RA	[167], 168], 181], 182]
			pSS	[174], 175]
			SLE	[169], [170], [171], [172]
			SSc	[165], 173]
		Baricitinib	RA	[181], [182], [183]
			pSS	[185], 186]
			SLE	[134], [178], [179], [180]
			SSc	[184]
		Upadacitinib	RA	[181], 182]
			AS	[190], [191], [192]
			pSS	[193], 194]
			SLE	[170], 195]
		Filgocitinib	RA	[181], 182], 196], 197]
			SLE	[195]
CTLA-4 agonists	By activating the CTLA-4 signaling pathway on regulatory T cells, thereby enhancing their inhibitory function on effector T cells, immune tolerance is reestablished.	Abatacept	RA	[199], [200], [201], [202], [203], [204], [205], [206], [207]

### 4.1 Cytokines and receptors

#### 4.1.1 Low-dose IL-2

A member of the IL family, IL-2 is produced by activated T cells. It stimulates T cell growth, proliferation, and differentiation by binding to the IL-2 receptor on the cell surface. Treg cells are important modulators of peripheral and lymphocyte tolerance because they have high-affinity receptors, which make them extremely sensitive to IL-2 in contrast to other T cells [109]. Treg cells can be activated by low-dose IL-2 without activating effector T cells, thereby mediating immune tolerance, suppressing autoimmune responses, and rebalancing the immune system [110]. Currently, low-dose IL-2 has garnered significant attention in the treatment of rheumatic and autoimmune diseases.

Patients with RA exhibit impaired Th17/Treg balance in their peripheral blood. Low-dose IL-2 therapy has been demonstrated to be safe and effective, significantly improving Treg cell level and function [111]. Studies have shown that after treatment, the absolute number of anti-inflammatory Treg cells increases threefold, while that of other CD4^+^ T cell subsets increases twofold, thereby correcting the effector T cell/Treg imbalance [112]. For patients with refractory (D2T) RA, low-dose IL-2 similarly alleviates this immune imbalance without significant side effects [113]. Patients with pSS exhibit reduced IL-2 levels in the peripheral blood and decreased absolute numbers of Treg cells [114]. Low-dose IL-2 therapy can restore the balance between Treg and Th17 cells, potentially by activating Treg cells to suppress Th17 cell production [115], 116]. Patients with SLE demonstrate lower IL-2 expression levels than healthy individuals, resulting in defective Treg cells [117]. Low-dose IL-2 therapy selectively modulates T cell function and level, correcting Treg cell defects caused by IL-2 deficiency [118]. Additionally, it regulates immune inflammatory responses by inhibiting Tfh and Th17 cell differentiation, aiding the body in restoring immune homeostasis. Relevant phase II clinical trials have confirmed its efficacy and safety in treating SLE, with potential to reduce infection rates. However, caution is warranted regarding its potential risk of exacerbating CD4^+^ T cell-mediated immunopathology [119]. In patients with AS, low-dose IL-2 therapy improves existing clinical symptoms, induces specific Treg expansion and activation, and does not activate effector T cells [120].

#### 4.1.2 IL-17/IL-17R inhibitors

Th17 cells’ main effector cytokine is IL-17A. The IL-17 family consists of at least six structurally similar members including IL-17A to IL-17F. These cytokines not only possess the ability to defend against extracellular pathogens but also participate in chronic inflammation and autoimmune diseases [121]. The homodimeric glycoprotein IL-17A is composed of amino acids and has a molecular weight of around 35 kDa. IL-17A exhibits varying degrees of homology with other family members, with the highest similarity to IL-17F *in vivo* [122], sharing 55 % homology. The two form a heterodimer via disulfide bonds to exert their functions [123], 124].

The recombinant fully human IgG1κ monoclonal antibody secukinumab targets IL-17A and prevents the spread of inflammation caused by IL-17A [125]. As an IL-17A antagonist, it targets IL-23/IL-17A to inhibit inflammation [126], 127]. Additionally, for the clinical management of moderate-to-severe plaque psoriasis and AS [126], 128], secukinumab has been authorized. Secukinumab promotes the proliferation and differentiation of chondrocytes in AS and accelerates cartilage repair and regeneration, thereby restoring normal spinal function [129]. Furthermore, a clinical double-blind trial showed that patients receiving 300 and 150 mg of secukinumab demonstrated a significant improvement in the Ankylosing Spondylitis Response Criteria 20 response, which greatly reduced the symptoms of psoriatic arthritis [130]. Additionally, secukinumab has been used in the clinical treatment of diseases such as RA [131], 132], SLE [131], SSc [133], and AS [134], 135].

Ixekizumab is another monoclonal antibody targeting IL-17A, used to treat psoriatic arthritis [136], 137] and RA [138]. The drug was developed by Eli Lilly in the United States and belongs to the IgG4 antibody class, selectively binding to the IL-17 receptor. In terms of safety, common adverse reactions include upper respiratory tract infections, oral candidiasis, and conjunctivitis [139], so ixekizumab should be used with caution in patients with chronic or recurrent infections. In RA and SLE [131], IL-17 suppresses the development and expansion of Th17 cells, thereby weakening the inhibitory function of Treg cells. In AS, IL-17 induces a high expression of IL-23, triggering an inflammatory response at attachment sites via γδT17 cells [135].

#### 4.1.3 IL-6/IL-6R inhibitors

By blocking Foxp3’s TGF-β regulation, IL-6 can lower Treg cell production. Furthermore, IL-6 triggers the development of naive CD4^+^ T cells into Th17 cells by activating the STAT3 signaling pathway, which inhibits Foxp3 and increases RORγt expression [140]. When IL-6 binds to the IL-6 receptor and the second transmembrane protein gp130, the IL-6 signaling cascade is started [141]. Additionally, IL-6 is a major contributor to the development of AS and is essential for immunological responses and activation in AS [142]. Currently, IL-6 receptor inhibitors, such as sarilumab and tocilizumab, exert their effects by inhibiting IL-6R through three distinct signaling pathways [143].

Tocilizumab, a recombinant humanized IgGI subclass monoclonal antibody that targets the IL-6 receptor, has been authorized by the European Medicines Agency (EMA) and the US Food and Drug Administration (FDA) for the treatment of RA [144]. Numerous trials have demonstrated that tocilizumab is safe and efficient in treating patients with RA who do not react well to TNF antagonists or other DMARDs [145]. Tocilizumab is a therapeutic option for people with active, refractory, or progressing RA because it efficiently lowers RA disease activity and enhances joint function [146]. It has also demonstrated good success in the clinical treatment of pSS [147], 148]. Additionally, tocilizumab significantly reduces disease activity in patients with SLE but has not been approved for treating SLE in major global markets [134], 149]. Research has validated the pathological mechanisms of AS, yielding negative phase III trial results; consequently, tocilizumab is neither approved nor recommended for treating AS [150]. Conversely, the drug significantly improves skin fibrosis in patients with early progressive SSc and remains the only biologic currently approved for treating adult SSc-associated interstitial lung disease [151], 152].

The FDA approved sarilumab, the first completely human monoclonal antibody that targets IL-6R [153]. In 2017, it was approved for marketing by the FDA. To date, the FDA, EMA, and National Medical Products Administration have all approved its use for treating adult patients with moderate-to-severe active RA who have an inadequate response to or intolerance to one or more DMARDs [154]. Blocking IL-6Rα with sarilumab is not an effective treatment for AS [155].

### 4.2 JAK inhibitors

JAK inhibitors are small-molecule targeted medications that disrupt the JAK/STAT signaling pathway by competitively inhibiting the phosphorylation of members of the JAK family (JAK1, JAK2, JAK3, and TYK2) [156], 157]. Numerous inflammatory and autoimmune illnesses have pathophysiological processes that include the JAK/STAT signaling pathway [158]. Twelve of the more than 70 JAK inhibitors now licensed for use as immunomodulators are used to treat autoimmune disorders [159]. Each JAK inhibitor exhibits key differences from others. The representative JAK inhibitors currently used in ARDs include tofacitinib (a JAK3 inhibitor), baricitinib (a JAK1/2 inhibitor), and the JAK1 inhibitors upadacitinib and filgotinib.

Tofacitinib is a non-selective JAK inhibitor that was the first to receive an FDA license for the treatment of RA. Furthermore, only 5 mg twice daily of tofacitinib is authorized for the treatment of patients with RA due to its ideal benefit-to-risk ratio [160], 161]. Research has demonstrated that tofacitinib can successfully lower the inflammatory factors that patients with SLE produce and reduce the expression of STAT4 and the rs7574865 allele, thereby inhibiting the occurrence of severe SLE [162]; it also reduces SLE activity, promotes the recovery of Th cell function, and enhances the cutaneous lupus erythematosus disease area and severity index [163]. Another study demonstrated the anti-inflammatory and anti-fibrotic effects of tofacitinib in SD [164]. TF is non-toxic to healthy cells and does not inhibit their JAK pathway; in disease states, TF remains in diseased cells and inhibits the progression of RA through the JAK/STAT pathway. A retrospective study found that tofacitinib was more effective than conventional immunosuppressants and glucocorticoids in treating skin-involved diffuse SSc [165]. Furthermore, combining TF with methotrexate (MTX) or other non-biologic DMARDs yields better treatment outcomes and fewer adverse reactions [166]. Extensive research has demonstrated that tofacitinib is effective in treating RA [167], 168], SLE [169], [170], [171], [172], SSc [173], and pSS [174], 175] among patients with arthritis-related dermatoses.

Baricitinib is the second JAK inhibitor approved for the treatment of RA [176]. It was created by Eli Lilly and Incyte Pharmaceuticals, and on February 13, 2017, the European Union approved its usage either by itself or in combination with MTX for patients with RA who had not responded well to or were intolerant of DMARDs [177]. Baricitinib inhibits IFN-γ-induced STAT1 phosphorylation in fibroblast-like synovial cells among patients with RA, thereby suppressing GLS/TP secretion, inhibiting angiogenesis, reducing inflammatory responses, and decreasing cartilage destruction. Additionally, it can downregulate cytokines regulating IFN in patients with SLE, such as chemokine ligand 10 and chemokine ligand 19 [178], and inhibit IFN-α, reducing the differentiation and maturation of podocytes in the kidneys and the expression of functional proteins, thereby improving lupus nephritis [179], 180]. According to a trial, baricitinib helps patients with SSc who have digital ulcers and skin fibrosis while delaying the progression of pulmonary interstitial fibrosis. The drug exerts broad-spectrum anti-inflammatory and immunomodulatory effects by blocking the JAK/STAT signaling pathway, thereby influencing intracellular signaling of multiple cytokines. It is indicated for the treatment of RA [181], [182], [183], SSc [184], pSS [185], 186], and SLE [134].

One of the second-generation drugs, upadacitinib is a highly selective inhibitor of JAK1, with 74 times the selectivity for JAK1 compared with JAK2 [187]. It was created by AbbVie Inc., and on August 16, 2019, the FDA authorized its usage as a monotherapy or combination treatment for adult patients with moderate-to-severe RA who do not respond well to MTX. Both JAK1/3-dependent cytokines (IL-2, IL-4, IL-15, and IL-21) and JAK2/2-dependent cytokines (IL-3 and GM-CSF) as well as the JAK2/TYK2-dependent cytokine G-CSF may be inhibited by upadacitinib [188]. The EMA has approved upatinib for the treatment of AS. Upatinib used orally among patients with active AS shows good treatment effectiveness. Oral administration of upatinib in patients with active AS reveals good therapeutic efficacy and tolerability, indicating that the drug can address the treatment demands of patients with active AS who have inadequate response to NSAIDs [189]. Additional studies have validated the clinical efficacy of upatinib in treating RA [181], 182], AS [190], [191], [192], pSS [193], 194], and SLE [170], 195].

Filgotinib is a selective JAK1 inhibitor that has demonstrated efficacy in multiple phase II clinical studies [196], 197]. It highly selectively inhibits JAK1, blocking the signaling of pro-inflammatory cytokines associated with JAK1 while avoiding or minimizing the inhibition of JAK2 and JAK3, thereby improving safety without compromising efficacy. Current phase II data indicate its efficacy and favorable short-term tolerability in RA, with particular potential advantages in hematologic safety [181], 182], 196]. Filgotinib has also demonstrated the ability to inhibit lupus-associated cytokine pathways in SLE [195].

### 4.3 CTLA-4 agonists

A fusion protein called abatacept combines the human IgG1 Fc fragment with the extracellular domain of CTLA-4. This efficiently blocks the connection between CD28 and CD80/CD86, which consequently prevents T cell activation [198]. Abatacept’s safety in patients with RA has been verified by numerous international randomized clinical trials [199], 200]. Abatacept is safer for older patients with RA than other biologics such as TNF-α inhibitors or IL-6 receptor antagonists [201]. B cells and T cells are more prevalent in the synovial fluid of patients with RA [202]. Abatacept is well tolerated by patients with RA and has been demonstrated to markedly reduce clinical symptoms [203], [204], [205], [206], [207], [208].

## 5 Discussion

ARDs represent a class of chronic inflammatory diseases caused by the immune system attacking its own tissues. The dynamic equilibrium between the pro-inflammatory role of Th17 cells and the immunosuppressive function of Treg cells maintains normal immune function in the human body. Therefore, this study explored the origins and functions of Th17 and Treg cells, related ARDs, and drugs that control their differentiation, providing scientific theoretical basis and new insights for the prevention and treatment of ARDs. The study identified signaling pathways influencing Th17/Treg cell immune imbalance, including the JAK/STAT, PI3K/Akt/mTOR, and TGF-β/Smad pathways. Th17 cell characterization primarily relies on the classic markers IL-17 and transcription factor RORγt, while Treg cells are defined by IL-10 and transcription factor Foxp3. Extensive experimental and clinical studies have confirmed that cytokine network dysregulation disrupts Th17 and Treg cell differentiation and function during the progression of ARDs, playing a pivotal role in mediating inflammation and regulating autoimmunity [209]. In clinical studies, DMARDs have been shown to regulate Th17/Treg cell immune imbalance in treating diseases such as RA, pSS, SLE, AS, and SSc. Th17 and Treg cells serve as important indicators for disease severity and joint activity.

T cell differentiation is closely linked to metabolic states. Research has indicated that cytokines and microbial metabolites can guide T cells toward distinct metabolic pathways, profoundly influencing their differentiation outcomes. Metabolism not only supplies cellular energy but also participates in signaling pathways, affecting gene expression and cellular functions. Th17 cells favor glycolysis for rapid proliferation and effector function, primarily driven by the mTORC1 signaling pathway. Conversely, Treg cells prefer oxidative phosphorylation and fatty acid oxidation, supporting their long-term survival and regulatory role. Furthermore, microbial metabolites act as histone deacetylase inhibitors, influencing metabolic states while serving as metabolic substrates or modulators. During late stages of cellular differentiation, when external signals diminish, mature Th17 and Treg cells maintain their identities through stable epigenetic mechanisms. DNA methylation at specific gene loci, such as those encoding Rorc and Foxp3, influences transcriptional states – activating or silencing genes – a function critical for maintaining cellular differentiation [132]. Many epigenetic modifiers rely on metabolic intermediates as cofactors or substrates, further highlighting the close link between metabolic reprogramming and epigenetic regulation. α-Ketoglutarate serves as a cofactor for TET DNA demethylases, while acetyl-CoA is utilized for histone acetylation, demonstrating that metabolic states directly influence the formation and maintenance of the epigenetic landscape. Metabolic reprogramming and epigenetic regulation in T cells not only play vital roles in normal immune responses but also possibly serve as key drivers in the development of autoimmune diseases. Abnormal “epigenetic imprinting” in Th17/Treg cells may explain the persistence and memory characteristics of autoimmune diseases, offering novel insights for developing drugs that target the epigenome.

This study examined five rheumatic immune diseases, all exhibiting Th17/Treg imbalance, yet each with distinct characteristics and predominant pathogenic mechanisms reflecting different immunopathological cores (Table 4). AS and RA both highlight abnormal Th17 cell proliferation responses. AS features IL-23/IL-17-driven systemic axial skeletal inflammation [210], while RA primarily involves local joint functional inactivation and transformation, with Th17 cells acting as direct executors of tissue damage. SLE emphasizes a fundamental defect in Treg function [59], 211], characterized by a systemic decline in both Treg number and function, leading to partial loss of immunosuppressive capacity. Regarding pSS, some studies have suggested local infiltration and destruction by Th17 cells in the secretory glands [69], while others have proposed that it relates to defects in immune tolerance due to Treg functional exhaustion within systemic immunity. The imbalance in SSc is the most complex, reflecting a dynamic evolution from early Th17-driven inflammation to late-stage fibrosis associated with Treg abnormalities, where Treg cell roles may be more critical [95]. These mechanistic differences explain why therapeutic agents targeting the same immune balance axis yield markedly divergent outcomes across diseases, suggesting that future immunomodulatory strategies must be tailored to disease-specific imbalances. The distinct equilibrium between Th17 and Treg cells in ARDs provides novel rationale for targeted therapeutic approaches.

**Table 4:** Comparison of Th17/Treg imbalance properties in ARDs.

Disease	Mechanism of imbalance	Th17 response	Treg response	Clinical/pathological association
RA	Primarily characterized by enhanced Th17 responses, supplemented by Treg dysfunction and loss of stability.	Active, produces IL-17, driving activation of fibroblastic synovial cells and bone erosion.	The number may be normal or even increased, but regulatory function is severely impaired, and they readily convert to Th17-like cells in inflammatory environments.	High expression of pathogenic genes in Th17 cells and dysfunctional Tregs fail to suppress persistent intra-articular immune responses, leading to chronic synovitis and bone destruction.
AS	Primarily driven by the Th17/IL-23 axis, with relatively limited evidence of Treg defects.	IL-23R gene polymorphism strongly correlates with disease; IL-23 drives sustained Th17 activation in axial joints/tendon insertions.	May be relatively deficient or functionally suppressed by the inflammatory environment.	Associated with new bone formation and bone erosion. Anti-IL-17/IL-23 therapy is highly effective.
SLE	Deficiencies in Treg numbers and function, accompanied by polyclonal, multi-organ-targeted Th17 and follicular helper T cell responses.	Active, contributes to organ damage in kidneys (lupus nephritis), skin, and other tissues.	Reduced numbers with diminished regulatory function. Inability to maintain tolerance toward reactive B cells and T cells.	The complete collapse of Tregs leads to systemic autoimmunity. This imbalance is highly correlated with multisystem involvement and autoantibody production.
pSS	Within target organs (salivary/lacrimal glands), dominated by Th17 infiltration and local inflammation, accompanied by restricted Treg function or their shift toward pathogenicity.	Highly infiltrates glands, directly causing glandular epithelial cell injury and suppressing glandular secretory function.	In the glandular microenvironment, Tregs may be insufficient in number or functionally suppressed.	Th17-mediated glandular destruction is the direct cause of core symptoms such as dry mouth and dry eyes. The imbalance is more confined to the affected exocrine glands.
SSc	Early stages may feature Th17-driven inflammation, while late stages correlate with fibrosis progression due to Treg dysfunction.	Early activation stimulates IL-17 and IL-21 secretion, activating fibroblasts and promoting collagen production.	May exhibit functional insufficiency or excessive suppression, leading to impaired anti-fibrotic immune responses and increased risk of chronic infection.	The imbalance between Th17 and Treg cells is closely associated with the transition from the inflammatory phase to the fibrotic phase. Abnormalities in Treg function may promote rather than inhibit the progression of fibrosis.

In rheumatic and immune-mediated diseases, the proportion of Th17 cells correlates positively with the inflammatory response associated with disease onset. The reduced suppressive function of Treg cells may result from an imbalance between Th17 and Treg cells or from damage to Treg cells caused by the disease itself. This finding not only confirms the role of Th17/Treg imbalance in rheumatic and immune-mediated diseases but also highlights two key points of contention within the current field. First, regarding Th17/Treg plasticity, some researchers have proposed that high levels of inflammatory mediators including IL-1β and IL-6 may drive Treg cells toward Th17-like differentiation, thereby exacerbating immune imbalance [212]. Our study found coexisting Treg functional decline and Th17 expansion, providing some support for this hypothesis. However, more refined lineage tracing studies are needed to directly validate this plasticity event. Second, another perspective suggests that in immune diseases, besides the pro-inflammatory role of Th17 cells, direct impairment of Treg cells may also lead to reduced Treg numbers, resulting in dual immune imbalance. This view has been confirmed in SSc [91]. In ARDs, the dominant mechanism involves pathogenic Th17 cells driving Treg conversion to create immune imbalance. However, the specific changes in the equilibrium between these two cell types have not yet been targeted. Future single-cell sequencing studies will help elucidate this, which is crucial for precision targeted therapy [213]. Therefore, our work not only describes the manifestations of imbalance but also raises new questions for further exploration of the underlying dynamic cellular fates and heterogeneity.

This study identified drugs with clinical potential for treating ARDs by modulating the Th17/Treg balance. To gain deeper insights into the causes of this imbalance and develop precision therapies, future research should focus on the following directions: First, at the mechanistic level, single-cell multi-omics technologies should be employed to map precise cellular and molecular networks responsible for Treg dysfunction and abnormal Th17 activation within disease-specific target organ tissues. In RA, focus should be placed on the impact of metabolic stress on Treg plasticity within synovial-specific niches, whereas in SSc, dynamic analysis of Treg functional transitions within fibrotic niches is essential. Second, at the translational level, developing strategies that specifically remodel the local immune microenvironment rather than inducing systemic immunosuppression is crucial. This includes targeted local administration to the tendon-end microenvironment in AS or the salivary glands in pSS. Finally, at the personalized level, future efforts should focus on establishing biomarkers capable of distinguishing patient subtypes driven primarily by Th17 activation versus Treg deficiency, thereby enabling personalized treatment based on the underlying imbalance mechanisms.
